# The impact of fascia iliaca nerve blockade on early postoperative pain and recovery after hip arthroscopy for femoroacetabular impingement syndrome

**DOI:** 10.1093/jhps/hnab076

**Published:** 2021-10-28

**Authors:** Grant August, Andrea H Johnson, Justin J Turcotte, Benjamin M Petre

**Affiliations:** Anne Arundel Medical Center Orthopedics, 2000 Medical Parkway, Suite 503, Annapolis, MD 21401, USA; Anne Arundel Medical Center Orthopedics, 2000 Medical Parkway, Suite 503, Annapolis, MD 21401, USA; Anne Arundel Medical Center Orthopedics, 2000 Medical Parkway, Suite 503, Annapolis, MD 21401, USA; Anne Arundel Medical Center Orthopedics, 2000 Medical Parkway, Suite 503, Annapolis, MD 21401, USA

## Abstract

Fascia iliaca nerve blockade (FIB) has been previously described as an effective technique for reducing postoperative pain and opioid consumption after hip arthroscopy for femoroacetabular impingement syndrome (FAIS). We hypothesize that an FIB will significantly reduce opioid consumption, pain scores and recovery time in our population. A retrospective observational study of 326 consecutive patients undergoing hip arthroscopy for FAIS at a single institution was performed. Patients were classified based on whether or not they received an FIB. Patient demographics, surgical details, medication details and 6-month postoperative outcomes were collected. The primary endpoint was the amount of narcotics required intraoperatively and in the postanesthesia care unit (PACU). Of the 326 patients included in the study, 37 received an FIB. No differences in sex, age or other surgical details were observed between groups. Patients receiving an FIB were more likely to receive celecoxib (*P* < 0.001), pregabalin (*P* = 0.001) and methocarbamol (*P* = 0.002). The FIB group received lower doses of narcotics intraoperatively (*P* = 0.001), postoperatively (*P* < 0.001) and in total (*P* < 0.001). The FIB group also self-reported lower first pain scores upon arrival to PACU (*P* = 0.001) and experienced shorter PACU recovery times (*P* < 0.001). After controlling for differences between groups, patients who received an FIB required significantly lower amounts of narcotics, had shorter PACU times and lower first PACU pain score than those who did not (*P* < 0.001). No differences in complication rates were noted between groups. The use of FIB resulted in lower pain scores, reduced recovery time and decreased early postoperative narcotic requirements for patients undergoing hip arthroscopy for femoroacetabular impingement. Further study is required to validate these findings and determine the optimal approach to regional analgesia in this patient population.

## INTRODUCTION

The incidence of hip arthroscopy procedures for treatment of femoroacetabular impingement syndrome (FAIS) has increased significantly since the mid-2000s. From 2006 to 2010, a 600% increase in procedure volume was observed in American Board of Orthopedic Surgery (ABOS) Part II examinees and the overall incidence increased from 3.6 per 100 000 in 2005 to 16.7 per 100 000 in 2013 [1, 2]. Hip arthroscopy has been demonstrated to be an effective treatment for FAIS, with one large study finding 87.7% of patients achieved to return to sport after surgery [3]. However, a challenge of these surgeries is achieving appropriate pain management while also decreasing opioid consumption during the postoperative period [4, 5]. Across a variety of orthopedic surgeries, including femoral neck fracture and hip and knee arthroplasties, neuraxial and regional anesthesia has been successfully used to manage early postoperative pain [6, 7].

For patients undergoing hip arthroscopy specifically, fascia iliaca nerve blockade (FIB) has been previously described as an effective technique for reducing postoperative pain and opioid consumption [8, 9]. FIBs primarily target the femoral nerve and the lateral femoral cutaneous nerve contained within the fascia iliaca compartment [10]. The purpose of this retrospective study is to evaluate whether FIB resulted in improved perioperative pain control in our population of patients undergoing hip arthroscopy for FAIS. We hypothesize that a pre-incision FIB will significantly reduce intraoperative and early postoperative opioid consumption, postanesthesia care unit (PACU) pain scores, and time spent in the PACU.

## METHODS

### Study population and setting

This study was deemed institutional review board exempt by the institution’s clinical research committee. A retrospective observational study of consecutive patients undergoing hip arthroscopy for FAIS was performed. All surgeries were performed by a single surgeon at a single institution from July 2018 to January 2021. All patients were confirmed to have a cam or pincer impingement that was surgically treated with bony resection along with repair or reconstruction of the labrum. Patients who were under 18 at the time of surgery, pregnant or prisoners were excluded.

### Perioperative protocol

All surgeries were performed on an outpatient basis under general anesthesia; regional anesthesia with FIB was performed at the discretion of the surgeon and anesthesiologist for postoperative pain control. Preoperative multimodal pain management was implemented for appropriate patients in March 2019 and consisted of celecoxib, pregabalin, and robaxin given in the preoperative holding area. Patients received some or all of these medications at appropriate dosages based on their individual medical histories. Intraoperative local anesthesia was used for all patients at the end of the case with deep infiltration into the joint space. Patients that did not receive an FIB were injected with 30 ml of 0.5% bupivacaine and patients that did receive an FIB were injected with variable amounts of 0.5% bupivacaine based on their weight and taking into account the amount of local anesthetic that they had already received with the block. All patients were subject to the same rehabilitation protocols with the exception of postoperative hip bracing, which was discontinued for most patients in October 2020. All patients began a supervised rehabilitation program during Postoperative Week 1. Initial range of motion (ROM) restrictions are as follows: hip flexion from 0–120 weeks 0–3, abduction 0–45 weeks 0–3 and no external rotation for 2 weeks. Strength and ROM with restrictions are progressed as per protocol. All patients are toe touch weight bearing with crutches from weeks 0 to 3 and then progress to weight bearing as tolerated and wean from crutches.

### Regional anesthesia protocol

Fascia iliaca blocks were performed by a select group of anesthesiologists with prior training in this technique. Blocks were performed using ultrasound guidance using the technique described in the New York School of Regional Anesthesia guidelines [11]. Patients received 30–50 ml of ropivicaine 0.25% with the dosage determined by patient weight. The FIB was administered in the OR under intravenous sedation, prior to the administration of general anesthesia.

### Data collection and analysis

Patient demographics and operative details were extracted from the electronic medical record using structured query language. Surgical details, pre-, intra- and postoperative medication details, 6-month postoperative outcomes, and patient-reported outcomes were collected by manual chart review. Patient-reported outcomes [Patient-Reported Outcomes Measurement Information System Global Health (PROMIS-GH) physical health and Hip Disability and Osteoarthritis Outcome Score Joint Replacement (HOOS JR)] were collected on a voluntary basis and not available on all patients. Lower extremity functional score (LEFS) was only available on patients who attended physical therapy through the hospital-based practice and thus not available on all patients. The primary endpoint was the level of narcotics required intraoperatively and in the PACU. Opioid consumption was converted to oral morphine milligram equivalents (OMME). In addition, preoperative multimodal pain medications and muscle relaxants received in the preoperative holding area were recorded so that they could be controlled for in multivariate analysis. Patients were then grouped based on whether or not they received an FIB, and univariate comparisons were performed using two independent sample *t*-tests for continuous variables and chi-square testing for categorical variables. The Fisher’s exact test was used in place of chi-square when the assumptions of chi-square testing were not met. A subgroup analysis comparing OMME across four categories of whether or not multimodal analgesia was used and whether or not a block was used was performed using one-way analysis of variance with *post hoc* Bonferroni correction to compare between-group differences. Multiple linear regression was then performed to evaluate whether the use of an FIB was associated with decreased OMME requirements after controlling for differences between the groups. All statistical analyses were performed in SPSS version 27 (IBM, Armonk, NY).

## RESULTS

Of the 326 patients included in the study, 289 (89%) did not receive an FIB and 37 (11%) received an FIB. No differences in sex or age were observed between groups, and patients receiving an FIB had lower body mass indexes (BMIs) on average (25.7 ± 4.4 vs. 27.7 ± 5.9 kg/m^2^, *P* = 0.044). No differences in other surgical details including the proportion of patients with American Society of Anesthesiologists Score (ASA) ≥ 3, reconstruction or bony resection type, or operative time were observed between groups (Table I).

**Table I.** Patient demographics and surgery details

*Variable*	*No block* *N * *= * *289*	*Block* *N * *= * *37*	*P-value*
Demographics			
Female, *n* (%)	109 (37.7)	11 (29.7)	0.343
Age, y (mean ± SD)	38.9 ± 13.2	38.4 ± 13.0	0.831
BMI, kg/m^2^ (mean ± SD)	27.7 ± 5.9	25.7 ± 4.4	**0.044**
Surgery details			
ASA ≥ 3, *n* (%)	34 (11.8)	3 (8.1)	0.782*
Labral repair, *n* (%)	250 (86.5)	34 (91.9)	0.445*
Labral reconstruction, *n* (%)	37 (12.8)	3 (8.1)	0.595*
Cam resection, *n* (%)	287 (99.3)	37 (100.0)	1.000*
Pincer resection, *n* (%)	179 (61.9)	28 (75.7)	0.102
OR time, min (mean ± SD)	126.7 ± 27.6	121.1 ± 21.8	0.239

P-values < 0.05 in bold.

* Indicates use of Fisher’s exact test.

OR, operating room (wheels in to wheels out).

Evaluation of multimodal analgesics used preoperatively found no difference in rates of oral acetaminophen use between groups. However, patients receiving an FIB were more likely to receive celecoxib (78.4 vs. 46.4%, *P* < 0.001) and pregabalin (81.1 vs. 53.3%, *P* = 0.001). Similarly, a higher proportion of patients in the block group received preoperative methocarbamol (81.1 vs. 54.7%, *P* = 0.002) (Table II).

**Table II.** Preoperative multimodal pain medication use

*Variable*	*No bloc* *k* *N * *= * *289*	*Bloc* *k* *N * *= * *37*	*P-value*
Oral acetaminophen, *n* (%)	4 (1.4)	0 (0.0)	1.000*
975 mg	3 (75.0)	N/A	N/A
Celecoxib, *n* (%)	134 (46.4)	29 (78.4)	**<0.001**
100 mg	100 ± 0	100 ± 0	N/A
Pregabalin, *n* (%)	154 (53.3)	30 (81.1)	**0.001**
75 mg	39 (25.3)	14 (46.7)	**0.018**
25 mg	112 (72.7)	16 (53.3)	**0.035**
Methocarbamol, *n* (%)	158 (54.7)	30 (81.1)	**0.002**
750 mg	147 (93.0)	30 (100.0)	0.136

*P*-values < 0.05 in bold

* Indicates use of Fisher’s exact test.

In comparison to the no-block group, patients receiving an FIB received lower doses of narcotics intraoperatively (37.8 ± 15.1 vs. 50.9 ± 23.9 OMME, *P* = 0.001), postoperatively (16.8 ± 15.0 vs. 33.1 ± 20.4 OMME, *P* < 0.001) and in total (54.6 ± 19.6 vs. 84.1 ± 30.1 OMME, *P* < 0.001). Patients in the FIB group also self-reported lower first pain scores upon arrival to PACU (2.9 ± 3.4 vs. 4.9 ± 3.4, *P* = 0.001), but no difference was found when comparing the final pain score prior to discharge. PACU recovery times were also shorter in the FIB group (72.1 ± 32.7 vs. 118.1 ± 58.6 min, *P* < 0.001) (Table III).

**Table III.** Perioperative outcomes

*Outcome measure*	*No block* *N * *= * *289*	*Block* *N * *= * *37*	*P-value*
Intraoperative MME (mean ± SD)	50.9 ± 23.9	37.8 ± 15.1	**0.001**
Postoperative MME (mean ± SD)	33.1 ± 20.4	16.8 ± 15.0	**<0.001**
Total MME (mean ± SD)	84.1 ± 30.1	54.6 ± 19.6	**<0.001**
First PACU pain NRS (mean ± SD)	4.9 ± 3.4	2.9 ± 3.4	**0.001**
Last PACU pain NRS (mean ± SD)	4.2 ± 1.9	3.8 ± 2.6	0.279
PACU minutes (mean ± SD)	118.1 ± 58.6	72.1 ± 32.7	**<0.001**

*P*-values < 0.05 in bold.

NRS, numeric rating scale.

Table IV examines postoperative outcomes and complications between groups. There were no significant differences in number of complications in patients that received an FIB and patients that did not. No patients in the FIB group had a fall in the first 2 weeks postoperatively. Complications included two patients with superficial wound infections, one patient underwent repeat surgery for a retained surgical implement, seven patients had residual numbness on the operative side which ultimately resolved, one patient required a manipulation under anesthesia for decreased range of motion and one patient had a revision hip arthroscopy. Table V examines patient-reported outcomes during the perioperative period; there were no significant differences between patients in the FIB group compared with the group that did not have an FIB.

**Table IV.** Postoperative complications

*Outcome measure*	*No block* *N = 289*	*Block* *N = 37*	*P-value*
Fall 0–14 days, *n* (%)	4 (1.4)	0	1.000*
Any complication 0–30 days^a^, *n* (%)	12 (4.2)	0	0.374*
Any complication 31–90 days, *n* (%)	3 (1.0)	1 (2.7)	0.384*
Any complication 91–180 days, *n* (%)	2 (0.7)	0	1.000*
Any complication up to 6 months, *n* (%)	17 (5.9)	1 (2.7)	0.705*
90-Day ED return, *n* (%)	3 (1.0)	0 (0.0)	1.000*

*P*-values < 0.05 in bold.

* Indicates use of Fisher’s exact test.

ED, Emergency department.

a Includes falls from 0 to 14 days.

**Table V.** Perioperative patient-reported outcomes

*Outcome measure*	*No block* *N = 289*	*Block* *N = 37*	*P-value*
Preoperative PROMIS-GH physical health (mean ± SD)^a^	42.41 ± 7.79	44.43 ± 7.92	0.310
Last postoperative PROMIS-GH physical health (mean ± SD)^b^	45.08 ± 9.03	50.10 ± 10.03	0.074
Preoperative HOOS JR (mean ± SD)^c^	59.60 ± 13.14	59.09 ± 13.26	0.880
Last postoperative HOOS JR (mean ± SD)^d^	73.18 ± 17.11	79.02 ± 15.14	0.217
First postoperative LEFS (mean ± SD)^e^	23.78 ± 24.20	20.42 ± 16.57	0.593
Last postoperative LEFS (mean ± SD)^f^	53.73 ± 14.94	50.86 ± 17.33	0.639

a 102 patients.

b 68 patients.

c 104 patients.

d 66 patients.

e 75 patients.

f 62 patients.

In the subgroup analysis, patients were grouped into one of four categories: no block, no multimodal analgesia; no block with multimodal analgesia; block, no multimodal analgesia; and block with multimodal analgesia. Across these four groups, a significant difference in OMME requirements was observed (*P* < 0.001) (Table VI). No between-group differences were observed between patients not receiving a block with or without multimodal analgesia (*P* = 0.058) or between patients who received a block with or without multimodal analgesia (*P* = 1.000). However, patients receiving a block without multimodal analgesia and with multimodal analgesia all received less OMME than patients in the no-block groups (all *P* < 0.05). After controlling for differences between groups—BMI, celecoxib, pregabalin and methocarbamol usage, patients who received an FIB required significantly less OMME (β = −25.371, *P* < 0.001), had significantly lower first PACU pain scores (β = −1.963, *P* = 0.001), and spent less time in PACU (β = −41.680, *P* < 0.001) than those who did not (Table VII).

**Table VI.** Univariate comparison of OMME by the use of multimodal analgesia and block

*Outcome*	*No block, no multimodal (N = 161)*	*No block, multimodal (N = 128)*	*Block, no multimodal (N = 10)*	*Block, multimodal (N = 37)*	*P-value*
OMME received	88.0 ± 30.6	79.1 ± 28.8	53.0 ± 20.4	55.3 ± 19.7	**<0.001**

*P*-values < 0.05 in bold.

**Table VII.** Multiple linear regression analysis of postoperative outcomes after controlling for differences between groups

*Variable*	*Unstandardized β*	*95% Confidence interval*	*P-value*
Outcome: Total OMME consumption			
BMI	1.084	0.534 to 1.635	**<0.001**
Celecoxib	−2.845	−14.247 to 8.557	0.624
Pregabalin	−10.383	−27.497 to 6.731	0.234
Methocarbamol	7.251	−10.183 to 24.686	0.414
Fascia iliaca block	−25.372	−35.420 to −15.325	**<0.001**
Outcome: First PACU pain score			
BMI	−0.005	−0.071 to 0.061	0.881
Celecoxib	−0.666	−2.029 to 0.696	0.337
Pregabalin	−1.055	−3.100 to 0.989	0.311
Methocarbamol	1.734	−0.349 to 3.817	0.102
Fascia iliaca block	−1.963	−3.163 to −0.763	**0.001**
Outcome: Minutes in PACU			
BMI	1.190	0.106 to 2.275	**0.032**
Celecoxib	−12.148	−34.611 to 10.315	0.288
Pregabalin	−7.838	−41.554 to 25.878	0.648
Methocarbamol	15.533	−18.815 to 49.880	0.374
Fascia iliaca block	−41.680	−61.475 to −21.886	**<0.001**

*P*-values < 0.05 in bold.

## DISCUSSION

The use of FIB in patients undergoing hip arthroscopy surgery was associated with reduced early postoperative opioid consumption, decreased postoperative pain scores and decreased time spent in the PACU in our patient population. Of note, the reduction in recovery time of 41 min, the reduction in first PACU pain score by almost 2 points and the reduced opioid requirement of over 25 OMME after risk adjustment are of particular operational and clinical significance. We also found no increase in postoperative complications, specifically no falls in the patients that received a block, and no differences in patient-reported outcomes between groups. Based on these findings, we suggest that FIB can successfully be incorporated into perioperative protocols for patients undergoing hip arthroscopy for FAIS and may improve the early recovery process.

The findings of this study are consistent with several prior studies supporting the efficacy of FIBs. A retrospective study including 716 patients who underwent hip arthroscopy found that PACU time and opioid consumption were reduced due to a supra-inguinal FIB, but pain scores did not reach statistical difference [12]. Another small randomized control trial evaluating the efficacy of FIBs in hip arthroscopy found FIB use to result in low opioid consumption and high-quality pain relief after surgery. Thirty subjects were included, of whom 6 required only oral opioids, 2 required only IV opioids, 20 required both oral and IV opioids, and 2 required no opioids while in recovery [8]. FIBs appear to have high efficacy with regard to reducing opioid consumption, PACU time and pain relief. Across all of the studies, FIBs were combined with multimodal analgesia techniques (including the use of fentanyl, celecoxib, gabapentin, etc.). This trend was consistent with our study, as we observed significantly higher rates of multimodal analgesia use in patients receiving an FIB. While this introduces potential confounding variables, a strength of our study is that a significant reduction in perioperative narcotic requirements was observed after controlling for the use of multimodal analgesia and muscle relaxants.

Although our findings add to a growing body of literature supporting the use of FIBs, FIBs are not a standard of care for hip arthroscopic procedures. Other authors have questioned the efficacy of regional anesthetics. One study found that FIBs had no statistical difference with regard to pain and patient satisfaction between an FIB group of 27 subjects and a no-block group of 33 subjects. Pain scores were recorded 1, 2, 4 and 7 days following surgery [13]. Similarly, a study comparing FIB in 41 patients and an intra-articular injection of local anesthetics in 43 patients concluded that there were no significant differences between morphine equivalent use of opioids and PACU recovery time. Pain scores were evaluated both while in the PACU and at routine check-up appointments 2 weeks, 6 weeks and 3 months postoperatively [14]. A recent systemic review compared five prospective studies evaluating the FIB against either another form of pain control or placebo and found that the FIB was not superior to other pain control methods [15]. These results are in contrast with the findings of the current study and indicate a need for further research about the efficacy of FIBs in patients undergoing hip arthroscopy surgery. While the above studies concluded that long-term pain reduction was not significant, our study indicates that FIBs are efficacious during early pain management while not increasing the postoperative complication rate. In comparison to these studies, a limitation of the current study is that evaluation of pain and narcotic consumption was only conducted in the PACU setting. However, our inclusion of a larger series of patients, particularly in the no-block group, is a strength.

Alternative regional anesthesia methods that have been described for patients undergoing hip arthroscopy include lumbar plexus, quadratus lumborum (QL) and pericapsular nerve group (PENG) blocks. In a study comparing the efficacy of an FIB group (25 subjects) to a lumbar plexus block group (23 subjects) following hip arthroscopies, FIBs were found to be easier to learn and implement and were more effective at reducing postoperative pain [16]. In a recent prospective, randomized, double-blind study by Haskins *et al.* comparing QL blocks and multimodal analgesia to multimodal analgesia alone, no significant difference was found between groups [17]. Another recent prospective study comparing FIB to the QL block found that the FIB group had a somewhat lower morphine consumption and the QL group had improved quadriceps strength in the early postoperative period [18]. Originally developed for and shown to be effective in controlling pain after surgical treatment of hip fractures, PENG blocks have recently been described in patients undergoing hip arthroscopy [19–21]. However, in a randomized controlled trial comparing 32 patients undergoing hip arthroscopy with lumbar plexus block and 32 patients with PENG block, no difference in PACU pain scores, narcotic requirements or recovery time was observed. No adverse events were observed in either group. Because the PENG block can be performed by the surgeon, it may facilitate increased OR efficiency while delivering comparable analgesia to the more technically demanding lumbar plexus or QL blocks [17, 22]. In alignment with the findings of Scanaliato *et al.*, the results of the current study support the assertion that FIB and PENG blocks hold promise as effective methods of perioperative pain control in patients undergoing hip arthroscopy [22]. Future study at our institution comparing the outcomes of these two regional anesthetic methods is underway.

There are multiple limitations of the current study. As a single-institution, single-surgeon observational cohort study with no *a priori* power analysis performed, it is possible that our population of patients is not representative of the broader population of patients undergoing hip arthroscopy for FAIS. Second, there is potential that selection bias exists as utilization of FIBs was at the discretion of the anesthesiologist in consultation with the patient. Third, other confounding factors may have influenced early postoperative outcomes including psychosocial factors which were not accounted for in this study. Although we attempted to control for these factors using multivariate methods, especially differences in the use of multimodal pain management medications, there is potential that other undocumented factors influenced pain and recovery.

## CONCLUSION

The use of FIB resulted in lower pain scores, recovery time and early postoperative narcotic requirements for patients undergoing hip arthroscopy for FAIS. Further study is required to validate these findings and determine the optimal approach to regional analgesia in this patient population.

## Data Availability

The data underlying this article will be shared on reasonable request to the corresponding author.
